# Small Talk: On the Possible Role of Trans-Kingdom Small RNAs during Plant–Virus–Vector Tritrophic Communication

**DOI:** 10.3390/plants12061411

**Published:** 2023-03-22

**Authors:** Emilyn E. Matsumura, Richard Kormelink

**Affiliations:** Laboratory of Virology, Wageningen University and Research, 6700 AA Wageningen, The Netherlands; emilyn.matsumura@wur.nl

**Keywords:** Small RNA-based communication, plant viral diseases, virus–plant interactions, plant viruses, trans-kingdom RNA silencing

## Abstract

Small RNAs (sRNAs) are the hallmark and main effectors of RNA silencing and therefore are involved in major biological processes in plants, such as regulation of gene expression, antiviral defense, and plant genome integrity. The mechanisms of sRNA amplification as well as their mobile nature and rapid generation suggest sRNAs as potential key modulators of intercellular and interspecies communication in plant-pathogen–pest interactions. Plant endogenous sRNAs can act in cis to regulate plant innate immunity against pathogens, or in trans to silence pathogens’ messenger RNAs (mRNAs) and impair virulence. Likewise, pathogen-derived sRNAs can act in cis to regulate expression of their own genes and increase virulence towards a plant host, or in trans to silence plant mRNAs and interfere with host defense. In plant viral diseases, virus infection alters the composition and abundance of sRNAs in plant cells, not only by triggering and interfering with the plant RNA silencing antiviral response, which accumulates virus-derived small interfering RNAs (vsiRNAs), but also by modulating plant endogenous sRNAs. Here, we review the current knowledge on the nature and activity of virus-responsive sRNAs during virus–plant interactions and discuss their role in trans-kingdom modulation of virus vectors for the benefit of virus dissemination.

## 1. Introduction

The gene-expression regulatory activities of non-coding small RNAs (sRNAs) are well-documented for a wide range of eukaryotic organisms, including plants [1,2,3]. Plant sRNAs are generated by Dicer-like proteins (DCLs)-mediated processing, in which double-stranded RNA (dsRNA) is recognized and cleaved into initial sRNA duplexes of 18–30 nucleotides in length [2,3,4,5]. One of the two strands of the processed sRNA duplexes is eventually incorporated into Argonaute proteins (AGOs), forming a complex that targets specific messenger RNAs (mRNAs) by sequence complementarity and leads to translational arrest, or cleavage and degradation of the targeted mRNAs as part of the RNA silencing mechanism, also known as RNA interference (RNAi) [5].

The biological processes regulated by sRNAs depend on the nature of the target mRNAs. In plants, sRNAs are known to be involved with the regulation of developmental and physiological processes, genome reprogramming and integrity, responses to abiotic stresses, and defense against plant pathogens and pests [2,5]. Among all plant pathogens, viruses are well-known for activating sRNA-based responses, mainly due to the production of dsRNA, as replicative intermediate molecules, which are effective inducers of RNA silencing [6]. In fact, it is suggested that conserved gene regulatory pathways have evolved from an sRNA-based cellular defense mechanism against viruses and transposable elements [2]. Thus, sRNAs in plants are numerous and have a heterogeneous profile according to their different origin and biogenesis, as well as to the (patho)physiological state of the plant. 

In general, sRNAs generated in plants are divided into two major classes: microRNAs (miRNAs) and small interfering RNAs (siRNAs). Most miRNAs are plant endogenous sRNAs derived from RNA precursors with imperfect hairpin structures, while siRNAs are processed from long dsRNAs derived from different sources (endogenous or exogenous), such as inverted repeats, sense-antisense overlapping transcripts, or synthesis by RNA-dependent RNA polymerases (RDRs) [7,8]. Also depending on the biogenesis of the siRNAs, they can be further divided into different subcategories: trans-acting siRNAs (ta-siRNAs) or secondary phased siRNAs (phasiRNAs), natural antisense transcripts-derived siRNAs (nat-siRNAs), heterochromatic siRNAs (hc-siRNAs), and long siRNAs (lsiRNAs) [3,4,7,8]. The sRNA categorization and their potential roles in plants have been extensively reviewed elsewhere [7,9,10,11,12]. In this review, we focus on sRNAs potentially involved in virus–plant interactions.

Plant virus diseases affect many important crops and lead to considerable economic losses in agriculture worldwide. By unraveling and understanding the virus-mediated alterations leading to plant disease development and/or plant resistance mechanisms, new strategies can be developed in order to fight plant viral diseases. In addition of modulating protein, volatiles, and phytohormones content, virus infection also significantly alters the accumulation and profile of sRNAs in infected plants, not only by generating virus-derived siRNAs (vsiRNAs) as a consequence of the activation of the antiviral RNA silencing pathway, but also for altering the profile of plant endogenous sRNAs [13]. Furthermore, as a defense mechanism against the plant antiviral response, most plant viruses encode for proteins that suppress the host RNA silencing activity by directly interfering with different steps of the pathway. These proteins are designated as viral suppressors of RNA silencing (VSRs), but they can also have side-effects on pathways of host endogenous sRNAs [13,14,15,16]. This already gives a hint on the substantial changes in the sRNAs profile of plants infected by a virus, as well as on the complexity of the sRNA-based communication during virus–plant interactions. Additionally, sRNAs have been found to be mobile, trafficking not only within an organism but also between interacting organisms to modulate gene silencing in trans [5,17]. This mechanism is termed as trans- or cross-kingdom RNAi and has been described as a key regulatory mechanism that also contributes to modulating the interaction between plants and different pathogens [8,18,19,20,21]. For instance, naturally occurring plant sRNAs can act in trans to silence pathogens’ mRNAs and impair virulence, while pathogen-derived sRNAs can target plant mRNAs and interfere with host defense [8]. Here, we give an overview of the roles of different virus-responsive sRNAs in virus–plant interactions and discuss the perspective of virus-mediated sRNA alterations modulating virus insect vector behavior for the benefit of dissemination. 

## 2. Virus-Derived Small RNAs as Modulators of Virus–Plant Interactions

Plants are exposed to a variety of conditions presented by their surroundings, such as abiotic and biotic stresses, that require physiological adjustments and/or immune responses that rely on their genome plasticity [13]. RNA silencing is one of the mechanisms helping plants to cope with these adjustments, by regulating gene expression in a highly specific manner due to the accumulation of specific sRNAs, which are the guiders and main effectors of gene silencing [13,14,15,16]. Thus, the (patho)physiological state of a plant is dependent on and, simultaneously, influences the profile of accumulated sRNAs.

As a result of the virus–plant co-evolution processes, plant viruses widely depend on their plant hosts for replication, protein expression, assembly, and spread throughout and between plants. This cycle of virus infection is accompanied by a variety of changes in the plant host as the result of differential gene expression and likely reflecting the general and specific interactions between the virus and the plant host [22]. General responses to virus infections include, for example, induction of pathogenesis-related (PR) and heat shock (HS) genes; the latter is likely the response to the accumulation of (misfolded) viral proteins in the cytoplasm [23,24,25]. Specific responses may include specific interactions between virus and plant proteins, but the majority of specific plant responses to virus infections involves RNA silencing, and therefore sRNAs.

When viruses infect susceptible plant hosts, virus-derived dsRNAs are made available (mostly as an intermediate product of virus replication) and trigger the activation of the host’s antiviral RNA silencing pathway. The virus-derived dsRNAs are cleaved by DCL proteins, producing vsiRNAs of 21–22 nts in size that direct sequence-specific and AGO-mediated posttranscriptional gene silencing (PTGS) of viral RNA. In case of DNA virus infections, 24 nts sized vsiRNAs are additionally produced, which direct the transcriptional gene silencing (TGS) of the viral DNA genome [4,13,26,27,28]. However, it has been shown lately that the contribution of RNA silencing to virus–plant interactions is highly complex and diverse across different virus–plant systems [4,13,26,29,30,31]. For instance, AGO loaded with plant vsiRNAs can target not only the viral RNA but also have the ability of targeting host mRNAs, leading to the modulation of host gene expression, either by directly targeting gene mRNAs or by interfering with the expression of host miRNAs that eventually reflect in changes to the expression of their targets [26]. This seems to be especially important in newly infected cells, when the virus needs to suppress the plant host’s defense and use cell components and functions to successfully establish infection [26,29,30,31,32,33]. Furthermore, sRNAs (signal of RNA silencing) can be amplified through plant RDR-mediated mechanisms and spread from the site of infection to mediate systemic silencing effects throughout the plant [1]. Thus, the accumulation of virus-responsive sRNAs in infected plants may lead to a coordinated modulation of communication between the virus and host plants.

### Virus-Derived sRNAs Regulating Plant Host Genes Expression

After the discovery of RNAi and its antiviral activity in 1990s, plant virologists started to focus on vsiRNA profiling, studying how viruses evade the cytoplasmic RNA degradation machinery or suppress antiviral RNAi, and to exploit RNAi by development of RNAi-mediated resistance strategies. Only a decade later, this view expanded more towards RNAi-mediated (modulating) effects on the host, caused either by the virus or their vsiRNAs, as beneficial towards the establishment and maintenance of virus replication and dissemination.

**Figure 1:**
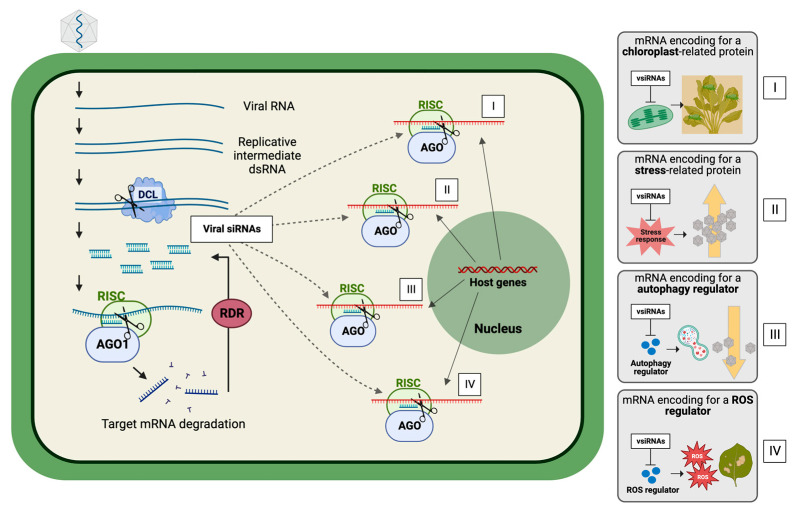
Implicated roles of virus-derived siRNAs (vsiRNAs) in plant–RNA virus interaction. During antiviral RNA silencing, generated vsiRNAs not only target the virus genome but can also modulate the expression of host genes. In the case of RNA virus infection (represented in this figure), viral replicative intermediate double-stranded RNAs (dsRNAs) are processed by Dicer-like proteins (DCL) into ~ 21 nucleotide (nt) primary vsiRNAs. These vsiRNAs can be loaded into AGO1 to form an active RNA-induced silencing complex (RISC) that mediates the silencing of the respective virus genome (left). Plant RDR proteins are involved in the generation of secondary vsiRNAs. Both primary and secondary vsiRNAs can be loaded into different AGOs for the amplification of RNA silencing. Plant virus-derived siRNAs may also act in trans to modulate the viral–host interplay when functional vsiRNAs, exhibiting complementarity to host genes, are loaded into the RNA silencing complex, change host gene expression and, consequently, affect different plant biological processes, depending on the nature of the host target mRNAs (panels at the right hand side). For example: (I) silencing of chloroplast-related genes by vsiRNAs may induce symptoms such as yellowing of the leaves, turning plants more attractive to insect vectors; (II) silencing of stress-related genes may lead to increased susceptibility of the host to virus infection; (III) silencing of important regulators of autophagy may activate the antiviral autophagy mechanism that inhibits viral infection; and (IV) silencing of ROS regulators may amplify ROS signaling that promotes cell death.

By using combinatorial genome-wide-based approaches to study the virus-derived sRNA regulatory network in plants, several works have demonstrated that plant virus-derived sRNAs are able to modulate host gene expression by targeting a wide range of host endogenous mRNAs [11,26]. However, it is still not fully uncovered whether the silencing of some host mRNAs is just a side-effect of random base-pairing between vsiRNAs and host mRNA, or whether it is a result of coordinated mechanisms modulated by the virus. The fact is that changes in the expression of certain genes influence virus–plant interactions, either by favoring virus infection or by favoring plant defense. So far, only very few works have demonstrated direct evidence of vsiRNAs targeting host genes, with implicated roles of virus–plant interactions. These are briefly described below, but also summarized in Table 1 and Figure 1.

One of the first examples is the chlorosis observed in plants after cucumber mosaic virus (CMV) infection in the presence of Satellite Y (a subviral non-coding RNA molecule often associated with CMV). This turned out to result from the silencing of magnesium protoporphyrin chelatase I subunit (CHLI) mRNA in the host *Nicotiana tabacum* by a SatY-derived vsiRNA, leading to a loss of chlorophyl and causing yellowing of leaves. Mutation of the corresponding SatY sequence led to a mismatch of the vsiRNA on the chlorophyl synthesis gene, and disappearance of the yellowing. Although this can easily be taken as a coincidence and classified as side-effect damage, yellowing of leaves attracts insect vectors and as such will contribute to the dissemination of plant viruses from plant to plant [32,33] (Figure 1, panel I). A more recent example was found with a tomato yellow leaf curl virus (TYLCV)-derived siRNA targeting a long-noncoding lncRNA molecule involved in tomato disease symptomatology [34].

A study on tobacco mosaic virus (TMV) showed that vsiRNAs derived from the 3′ end of the TMV genome (TMV-vsiRNA) showed high sequence complementarity to a *Nicotiana benthamiana* gene that encodes for the C2-domain abscisic acid (ABA)-related (CAR) 7-like protein (Nb-CAR7). Nb-CAR7 plays a critical role in ABA signaling, an important hormone in plant responses to abiotic stresses as well as to plant pathogens, including viruses. Consistently, Nb-CAR7-overexpressing plants show a decrease in TMV RNA accumulation, while dowregulation of Nb-CAR7 resulted in a higher TMV RNA accumulation [35] (Figure 1, panel II).

Chinese wheat mosaic virus (CWMV) also generates a vsiRNA that contributes to virus infection in the wheat host by targeting and silencing the gene encoding for the vacuolar (H^+^)-PPases (VPs). The VPs are regulators of active proton (H^+^) transport through membranes and play key roles in plant responses to abiotic stresses. However, accumulation of CWMV RNA1-derived sRNA in wheat has been shown to affect the concentration of H^+^ in wheat cells, thus maintaining the cytoplasm alkaline, and creating a more favorable environment for CWMV replication [36].

On the other hand, vsiRNAs can also target plant mRNAs that encode for negative regulators of plant defense pathways. In this case, virus sRNAs might activate defense mechanisms of the host plant that can impair its own infection. The rice stripe tenuivirus (RSV) RNA 4-derived sRNA (vsiRNA-4A), for example, targets and downregulates the eukaryotic translation initiation factor 4A (NbeIf4A) in *N. benthamiana*, resulting in symptoms of leaf twisting and stunting [37]. Interestingly, eIF4A inhibits the function of an important component of the antiviral autophagy mechanism (ATG5) in both rice and *N. benthamiana*. In the case of RSV, autophagy plays a role by degrading the viral silencing suppressor p3 protein [38]. Thus, silencing of eIF4A by RSV vsiRNA-4A induces autophagy that inhibits viral infection (Figure 1, panel III).

Another example is a vsiRNA derived from wheat yellow mosaic virus (WYMV) RNA1. WYMV-derived vsiRNA1 has been shown to activate wheat immunity by targeting and modulating the expression of the wheat thioredoxin-like gene (*TaAAED1*) which encodes for a negative regulator of reactive oxygen species (ROS) in the chloroplast. Thus, WYMV-derived vsiRNA1 activates host immunity via ROS signaling, which promotes cell death and regulates defense-related genes [39] (Figure 1, panel IV).

As with RNA viruses, DNA virus infection also activates antiviral RNA silencing but, in addition to 21 nt sized vsiRNAs, it also generates 24 nt sized vsiRNAs that mediate transcriptional gene silencing of the viral DNA genome. Both siRNA classes may be involved in the silencing of host genes, although the 24 nt sized vsiRNAs would then cause transcriptional gene silencing of host genes. So far, however, reports demonstrating this are lacking.

Furthermore, in the past few years, several studies focused on sRNA–mRNA targeting prediction have identified a myriad of plant host RNAs as potential targets of virus-derived sRNAs, leading not only to downregulation but also upregulation of host genes. As a consequence, different plant biological processes are predicted to be modulated, depending on the nature of the host target mRNAs. In general, host target genes are usually involved with basal host defense, stress responses, protein synthesis/turnover, chloroplast functions, cellular structure, development, cell metabolism, and pathways of host defense. Table 2 lists the plant biological processes mostly affected by vsiRNA-mediated gene deregulation.

The wide range of host target mRNAs indicates that virus-derived sRNAs are determinants in the outcome of plant–virus interactions [11]. Interestingly, vsiRNAs are not the only virus-derived sRNAs involved in host gene modulation. MicroRNAs (miRNAs) derived from plant-infecting viruses have been identified in a few instances showing host gene silencing activity and playing roles in the virus infection cycle [54,55]. The entry of viral nucleic acid into the nucleus is important for the production of its derivative miRNA, since miRNA-processing proteins are nuclear specific [42]. Thus, geminiviruses (nucleus-replicating DNA viruses) are potential candidates for production of virus-derived miRNAs (vmiRNAs). In fact, pre-miRNAs from the genomes of two begomovirus (family *Geminiviridae*), African cassava mosaic virus (ACMV), and east African cassava mosaic virus-Uganda (EACMV-UG), were identified targeting host plant (Jatropha curcas) genes involved in biotic response, metabolic pathways, and transcription factors [42]. However, since the majority of plant viruses have a RNA genome that rarely enters into the nucleus, it is speculated that plant RNA virus-derived miRNAs are uncommon [11]. Surprisingly, production of virus-derived miRNAs from RNA virus genomes has been reported for members of the family *Potyviridae*. Turnip mosaic virus (TuMV)-derived miRNAs, for example, were found to downregulate a host stress-responsive gene (HVA22D) in Arabidopsis [56]. Sugarcane streak mosaic virus (SCSMV)-derived miRNAs have been shown to mediate the silencing of genes involved in the plastidial isoprenoid biosynthesis pathway, resulting in reduced chlorophyll and carotenoids content in SCSMV-infected sugarcane leaves [44]. 

## 3. Virus-Activated Plant Endogenous Small RNAs as Modulators of Virus–Plant Interactions

The activation of antiviral RNAi during plant viral infections not only triggers the accumulation of vsiRNAs, but it is also accompanied by the production of plant endogenous sRNAs (including viral activated vamiRNAs and vasiRNAs) with modulating effects on the host to support the establishment and maintenance of virus infections or, in some cases, to activate antiviral host defense mechanisms [57]. Viral infections can alter plant endogenous sRNAs at the transcriptional level [58], or may interfere with their processing/biosynthesis [59].

One of the first identified pathogen-induced host endogenous siRNAs involved in the regulation of plant immunity/defense against the pathogen was reported during a study on the bacterial pathogen *Pseudomonas syringae* pv. *tomato* (Pst) DC3000 [60]. The reported nat-siRNAATGB2 is specifically induced by the effector avrRpt2 from Pst DC3000 and represses its antisense target PPRL (pentatricopeptide repeats protein-like) gene, a negative regulator of the (avrRst2-triggered) RPS2-mediated resistance response.

**Figure 2:**
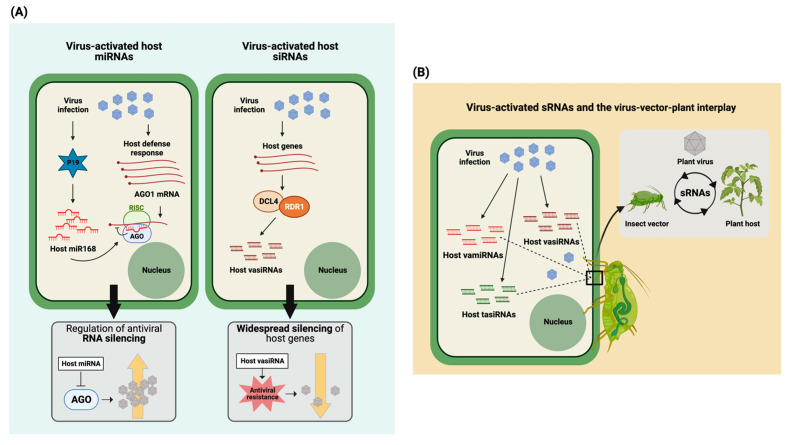
Implicated roles of virus-activated plant endogenous sRNAs in plant–virus interaction. In response to virus infection or activated by it, plants generate altered endogenous sRNAs profiles which modulate/fine-tune the expression of endogenous genes through RNA-mediated silencing. Besides the production of viral siRNAs, virus infection is accompanied by the accumulation of viral-activated host siRNA (vasiRNAs), microRNAs (vamiRNAs), and trans-acting siRNA (tasiRNAs). (**A**) This diverse group of plant sRNAs causes modulating effects on host genes that either support the establishment of virus infection or activate antiviral host defense mechanisms. Viral suppressors of RNA silencing (VSRs) play a role in modulating the accumulation and profile of host sRNAs. For example, the tombusvirus p19 VSR induces the overexpression of miR168, which arrests the translation AGO1-encoding mRNA, thus alleviating the antiviral RNAi pressure on virus replication (left panel). Plant RDRs also play roles in the production of vsiRNA. Production of virus-activated and RDR1-dependent siRNAs was observed to be involved with plant broad-spectrum resistance by widespread silencing of host genes (right panel). (**B**) Plant viruses rely on insect vectors for their dissemination, and during acquisition access feeding on virus-infected plants, the insect vectors may also acquire sRNAs. Since viruses utilize a plethora of strategies to increase their survival and spread, this tempts to hypothesize that during the modulation of plant sRNA profiles, some of these sRNAs are also transferred to insect vectors, where they modulate insect vector behavior for the benefit of virus dissemination (trans-kingdom RNAi). This hypothesis requires the examination of sRNA crosstalk up to the level of tritrophic interaction between virus, vector, and host. In case of viruses that are transmitted in a propagative manner (i.e., replicating in the insect vector as well), and not presented/explained in this figure, this could involve bi-directional trans-kingdom RNAi (virus-induced and altered sRNA profiles in both host plant and insect vector species).

The first studies on endogenous host sRNAs activated upon virus infection started to appear after the turn of the millennium in 2000. One of the first papers reporting on this phenomenon was from 2010, in which the induction of miRNA168 (the AGO1 regulator plant miRNA) by the tombusvirus p19 RNAi suppressor protein was shown [61]. Although p19 is able to bind siRNAs, it does not efficiently sequester miRNA168. While AGO1 is transcriptionally upregulated during viral infection, its targeting by miRNA168 leads to translational repression and, as a result, alleviates the antiviral RNAi pressure on tombusvirus replication (Table 3, Figure 2A, left panel). A study by Hu et al. [29] was one of the first to provide a comprehensive view on the global impact of a tobamovirus infection on the profile of host plant sRNAs. Changes in the profile of miRNAs and siRNAs, processed from ta-siRNA precursors, were observed upon oilseed rape mosaic virus (ORMV) infection. However, miRNAs and siRNAs whose levels were increased did not correlate to decreased levels of their target mRNA sequences and suggested that these virus-induced mi/siRNAs were sequestered or active at other/different levels. Furthermore, the study revealed a number of unique sRNAs derived from miRNA transcripts, that did not represent the known miRNAs. About half of these miRNA-like sRNAs (ml-sRNA) were also reported to be induced upon infection by the bacterial pathogen Pseudomonas (Table 3). In a study by Cao et al. [30], infection of Arabidopsis by CMVΔ2b (CMV with the 2b VSR ORF deleted) revealed an enhanced population of 21 nt sized endogenous host siRNAs that mapped to protein-coding sequences and comprised ~20% of the total population of siRNAs collected from virus-infected plants. In contrast to most siRNAs in Arabidopsis being RDR2- (24 nt siRNAs) or RDR6- (21 nt siRNAs) dependent, these designated viral-activated siRNAs (vasiRNAs) turned out to be RDR1-dependent and required AGO2 for their widespread silencing of target host genes. Production of these genetically identical vasiRNAs was also observed after challenging Arabidopsis with TuMV, a completely distinct virus, and led to a substantially overlapping set of host genes being targeted (Table 3). The induction of those vasiRNAs correlated with a resistance to both viruses, independently of viral siRNAs, and led to the idea that the induction of vasiRNAs provides a more broad-spectrum resistance by widespread silencing of host genes (Figure 2A, right panel) [30]. A second similar comprehensive study on vasiRNAs only appeared in 2021 [62], and identified ~15 loci in three different Brassica species from which vasiRNAs derived after challenging with cauliflower mosaic virus (CaMV). Interestingly, this class of vasiRNAs perfectly matched those identified by Cao et al. [30]. Another recent study by Fletcher et al. [63] also described the generation of vasiRNAs during infection of (susceptible) solanaceous hosts containing RDR1 with tomato spotted wilt and capsicum chlorosis orthotospoviruses (TSWV and CaCV, respectively), but not in the (susceptible) *N. benthamiana* LAB accession, known to lack a functional RDR1 gene. Abundant vasiRNAs were also absent from tomato variety Red Defender, containing the *Sw-5* resistance gene against orthotospoviruses. Many of the endogenous vasiRNAs identified in this study were similar to those identified in earlier studies [30,62], further strengthening the earlier raised idea of a common, more broad-spectrum resistance to viruses based on RDR1-dependent vasiRNAs.

From the vasiRNA-associated transcripts identified by Fletcher et al. [63], many were either encoding ribosomal proteins or proteins involved in protein processing and folding and located in the endoplasmic reticulum (ER). Previous studies by Guo et al. [64,65] revealed the identification of aminophospholipid transporting P-type ATPases 1 and 2 (ALA1 and -2), (membrane-bound) phospholipid flippases, and AVI2, a multispan transmembrane protein broadly conserved in plants and animals, and their role in the RDR1- and RDR6-dependent amplification of vsiRNAs and RDR1-dependent endogenous vasiRNAs. From these proteins, AVI2 is specifically induced upon CMV infection and is not required for the biogenesis of miRNAs and tasiRNAs, indicating a specific function in antiviral silencing [65]. As many RNA virus replication complexes reside in virus-induced vesicle-like membrane invaginations at membrane microdomains enriched with specific phospholipids, this tempted Guo et al. to propose the involvement of ALA1/ALA2 in the induction of similar invaginations for RDR-dependent production of dsRNA precursors of highly abundant viral and host siRNAs. This could explain the enrichment of vasiRNAs derived from transcripts associated with ER membranes, as suggested by Fletcher et al. [63]. Unless ER-membrane-associated transcripts have a major role in the regulation of defense pathways, it still remains unclear how vasiRNAs derived from these transcripts would provide a more broad-spectrum resistance against viruses.

Despite the limited number of studies, and first indications pointing towards a role for vasiRNAs in a broad-spectrum resistance against viruses, a possible pro-viral activity should not be ruled out, similar to what has been observed for cytoplasmic RNA processing bodies and stress granules that, for certain viruses, exhibit a pro-viral activity, but towards others function as anti-viral [66]. While the studies on vasiRNAs are only few, the number of studies pointing towards the identification and targeting of host genes by viral-activated endogenous host sRNAs (miRNAs/siRNAs) is likely to grow. Thus, further investigations are required to understand their roles in the virus disease cycle, which not necessarily need to be restricted to the interaction between the virus and host plant, but perhaps also reach to virus (insect) vectors, a third group of players that share intimate interactions with both of them and are required for virus dissemination (Figure 2B). 

## 4. Crosstalk between Virus and Insect Transmission Vector: Antiviral Defense Meets Herbivore Defense in Host Plant

Viral infections are known to cause a range of physiological and metabolic changes in the host plant, which are observed to influence the behavior and fitness of insect vectors as well [70,71]. A number of studies have shown a role of plant volatile emission spectra in the behavior of insect viral vectors and, consequently, in the transmission of the vectored viruses. Plants infected with a virus are preferred by the insect vector over healthy plants. This preference/early attraction relates to a combination of disease symptomatology (for example, yellowing of the leaves attracts insects) and changed volatile emission spectra [72]. Then, viruliferous insect vectors prefer healthy plants over virus-infected plants. The latter change again may be caused by, e.g., ongoing changes in the volatile spectrum leading to final deterrence. However, whether insect feeding behavior is simply a matter of attraction and deterrence is unknown, leaving the underlying mechanisms leading to changed vector behavior still to be fully explored. A role of viruses in this insect feeding preference is not unlikely, but mechanistic details are lacking. Several studies have indicated altered insect behavior due to viral infection, e.g., baculovirus infection of caterpillars causes a tree-top disease. Caterpillars normally move downwards into the soil for pupation, but upon baculovirus infection they move upwards. Limited studies indicate a role of viral genes and genes of the Lepidoptera species in altering insect behavior [73,74,75]. With plant viruses, alterations in vector behavior have been observed that may differ between viral infections that are transmitted in different modes (e.g., persistent CaMV versus non-persistent TuMV) [76], mostly involving, and, in several cases, even depending on the host plant [77]. Changes in vector behavior may also be induced by viruses that are not transmitted by the vector being studied [78]. In other studies, altered physiology/transcriptomes have been observed in aphids upon feeding on virus-infected plants, indicating the possible involvement of infected plant components in altered insect vector behavior [79,80]. Upon the feeding of insect vectors on virus-infected plants, pierce-sucking insects such as aphids, whiteflies and thrips inject saliva containing many effector proteins to suppress plant defense responses and prepare for feeding acquisition. One of the rapid changes monitored upon insect/herbivore feeding is the transient rise in cytosolic Ca^2+^ concentration and sensing by calmodulins (CaMs) or calmodulin-like proteins (CMLs) (Ca^2+^ sensors), to induce/activate downstream defense responses and a rapid increase in phytohormones such as jasmonic acid (JA), to defend and protect against herbivores. For further details on this topic, readers are referred to some recent reviews presented by Ghorbel et al. [81], Mostafa et al. [82], Parmagnani and Maffei [83], and Stroud et al. [84]. Upon herbivore attack, genes coding for these CaMs have been observed to be highly co-overexpressed with several endoplasmic reticulum-type Ca^2+^-transporting-ATPase genes, known to be involved in Ca^2+^ transport, indicating the importance of the Ca^2+^ signaling cascade in defense against insect attack [85,86]. 

Ca^2+^-ATPases act as ion transporters across cellular membranes and belong to the P-type ATPases superfamily, that either localize at the ER or at the plasma membrane [82]. Intriguingly, ER type, P-type ATPases were also identified as involved in RDR1 and RDR6 dependent amplification of vsiRNAs and RDR1-dependent endogenous vasiRNAs [64,65]. However, whether the ER type, P-type ATPases from the above studies are the same ones as earlier identified by Guo et al. [64,65] is unknown.

A recent study by Wang et al. [87] has shown that wounding, mimicking insect feeding, induces the elevation of calcium fluxes, which triggers calmodulin-dependent activation of calmodulin-binding transcription activator-3 (CAMTA-3). The latter protein activates RDR6 and bifunctional nuclease-2 (BN2), leading to priming and enhanced antiviral RNAi against geminiviruses, cucumoviruses, and potyviruses [87]. The earlier findings on the role of P-type ATPases in Ca^2+^ transport as part of a signaling cascade to defend against herbivores, but also in RDR1 and RDR6 dependent amplification of vsiRNAs and RDR1-dependent endogenous vasiRNAs [30,64,65,85,86], altogether strongly indicates a crosstalk and modulation/priming of herbivore and antiviral RNAi defense responses by Ca^2+^ signaling. 

Viruses do not only counterdefend against antiviral RNAi by direct interference at major steps of the RNAi pathway via viral suppressor proteins of RNAi (VSRs), topics that have been extensively reviewed in the past decade [15,28,88]. They are also able to dampen the Ca^2+^-induced, and further downstream, JA-mediated defense responses via the very same VSRs or other viral proteins [89,90,91,92,93], and as a result often enhance viral infection and modulate vector behavior/performance. For example, geminivirus V2 protein disrupts CAMTA3-calmodulin interactions [87]. The tobacco mosaic virus coat protein [lacking a (reported) silencing suppressor activity] inhibits calmodulin-like protein 30 (CML30) via an interacting L protein [94]. The TSWV NSs VSR protein, besides binding siRNAs, miRNAs, and long dsRNA, also binds calmodulin [95,96,97]. On the other hand, the βC1 silencing suppressors of tomato yellow leaf curl China betasatellite (TYLCCNB) and cotton leaf curl Multan betasatellite (CLCuMB), have been shown to respectively upregulate the expression of calmodulin or directly bind to it. Further studies on TYLCCNB revealed that calmodulin mediated the βC1 function in suppression of silencing, by repressing RDR6-dependent siRNA amplification [98,99]. A study by Anandalakshmi et al. [100] demonstrated an interaction between calmodulin and the tobacco etch virus HC-Pro silencing suppressor protein, but also the functionality of calmodulin as a silencing suppressor when expressed from a potato virus X vector. Calmodulin has also been shown to bind and direct the degradation of clover yellow vein virus (ClYVV) HC-Pro and CMV 2b. A study by Jeon et al. [101] furthermore demonstrated that induction of a systemic acquired resistance (SAR) response by salicylic acid mediates CMV 2b recognition and degradation by calmodulin. These studies only further strengthen the idea of intertwined antiviral and herbivore defense responses, in which suppression of a herbivore response could serve to support virus dissemination that relies on insect vector transmission. 

## 5. Viruses as Modulators of sRNA-Based Communication between Vectors and Host Plant, with Implications on Virus Spread

While nowadays RNAi is a commonly recognized antiviral defense mechanism involving the production of vsiRNAs, the accessibility to more affordable high-throughput sequencing technologies and metagenomics studies slowly help us to unveil a view in which viruses modulate another part of the sRNA spectrum that goes beyond vsiRNAs, i.e., the induction of host vamiRNAs and vasiRNAs. Not only are the effects of these sRNAs on viral disease and host defense responses yet to be fully uncovered, but also the absolute role of VSRs in suppression of the antiviral RNAi response, the modulation of host gene regulation and the ability to indirectly modulate other pathways, such as JA, that protect and defend against herbivores, via interaction/inhibition of factors such as calmodulin Ca^2+^ -sensors. Vice versa, insects feeding on host plants leads to direct changes in Ca^2+^ concentrations, and their sensing by calmodulins not only activates downstream defense responses, but also simultaneously activates RDR6 and bifunctional nuclease-2 (BN2) via transcription factors, culminating with the priming and enhancing of antiviral RNAi responses. So far, the activity of sRNAs produced in plants (either in the absence of a virus or induced upon virus infection) is considered to be primarily restricted to the host in which they arise, while viruses have also evolved an intimate relation with their vectors, such as aphids, whiteflies, and thrips, for dissemination. Since viruses utilize a plethora of strategies to manipulate the host cell machinery for the benefit of replication, it is very well possible that during the modulation of the plant sRNA profile some of these sRNAs also serve to modulate the host as well as the insect vectors regarding their attraction and feeding behavior, respectively, to enhance virus dissemination. Although there is no evidence for this concept yet, this idea is not unlikely considering the intimate tritrophic interactions between virus, plant, and vector and observed changes in vector feeding behavior.

What would support the idea of trans-kingdom sRNA communication that possibly might affect virus vector behavior and support virus spread? Which indications are there pointing to this? One of the first examples of trans-kingdom RNAi came from studies in which nematodes were fed with *E. coli* expressing dsRNAs [102], and demonstrated that RNAi signals are mobile and non-cell-autonomous, and can even move between different organisms and kingdoms. This phenomenon slowly has become known as host-induced gene silencing (HIGS).

Meanwhile, several examples of trans-kingdom RNAi can be found in plant–fungal pathogen interactions. For an overview on this subject, readers are referred to reviews from Hua et al. [103] and Chalone et al. [104], in which examples are described showing trans-kingdom sRNAs targeting mRNAs in both directions. Fungi have also been found to encode effector proteins that exert RNAi suppressor activity, so logically these may hinder bidirectional trans-kingdom RNAi and compromise the function of small, noncoding RNA effectors in host plant defense or in the colonization of the host by the pathogen. Whereas in the past decades, protein-based fungal effector translocation and effector-triggered immunity (ETI) in plant–fungi interactions have already been extensively studied and described, the mechanisms underlying trans-kingdom sRNA-regulated modulation of plant host and fungal pathogen still remain less studied.

Crosstalk between insect pests and other species (microbes, plants) at the level of protein or RNA effectors that affects virus vector competency and transmission has been observed. One of the first studies of a trans-kingdom protein effector to influence virus vector transmission identified GroEL, a protein produced by the endosymbiont of the aphid *Myzus persicae* and required as a chaperone to support (persistent) potato leaf roll luteovirus transmission [105]. In relation to RNA effectors, dsRNA-based insecticides have been designed and shown to be effective in combatting, e.g., the Colorado potato beetle in natural field situations [106] when plants are sprayed with a dsRNA formulation. The strategy to trans-activate RNAi also works when dsRNA is expressed in a transgenic plant and ingested by the Colorado beetle upon feeding [106], and altogether shows that insects can be affected by ingestion and uptake of dsRNA from external sources. In another very recent study, a long-noncoding RNA lncRNA molecule from the endosymbiont Wolbachia was found to activate anti-dengue defense pathway in *Aedes aegypti* mosquitoes, indicating the possibilities of ncRNA communication and modulation of virus insect vectors [107]. Findings such as these, and the important role of endosymbionts in the life cycle of insect pests and/or virus transmission, have also tempted researchers to elaborate on symbiont-mediated RNAi in aphids [108]. They also support the idea that sRNAs can be, and likely are, ingested by insect vectors during virus acquisition feeding on plants. However, reports on trans-kingdom RNAi/HIGS, involving either vsiRNAs, miRNAs, and/or vasiRNAs taken up from virus-infected leaf sources and targeting genes of the insect to subsequently modulate its behavior, still remain absent. Not only this remains elusive, but another big question on this process relates to the mechanism(s) on how these sRNAs reach/enter cells from the target insect vector. Whether such molecules, after insect feeding and entering the intestinal tract, are able to directly pass midgut epithelia barriers is not known. Although this remains speculative, another possible route/vehicle could be through extracellular vesicles that have received quite a lot of attention in the past decade and have been shown to enable intercellular communication via transport of small proteins, hormones, and sRNA molecules to (neighboring) target cells. While this area of research is booming in animal cell systems, research on this topic in plant systems is still in its infancy [12,109].

## 6. Perspectives

Despite the absence of reports on trans-kingdom RNAi between virus-infected plants and their virus insect vectors, a growing number of studies support the notion that during intimate tritrophic plant–virus–vector interactions, not only the host plant but also virus insect vectors are modulated by protein and sRNA (e.g., dsRNA) effectors, collected during virus acquisition feeding on infected plants, which ultimately affect insect behavior and virus competence for the benefit of virus dissemination. Considering that plant viruses are known to be transmitted by insects either non-persistently (stylet-borne), persistently (circulative via the insect body and salivary glands), or in propagative way (a mode of persistent transmission in which the virus also replicates in the insect), an additional challenge would be to find out whether insect vectors also are affected differently by trans-kingdom sRNAs, or if a similar/conserved set of trans-kingdom sRNA-mediated pathways are modulated in all these cases. Furthermore, whether vsiRNAs or miRNAs and vasiRNAs play a role during virus transmission by insects, or trans-kingdom RNAi is solely restricted to vasiRNAs, remains another interesting question waiting for scientific answer. In the occurrence of trans-kingdom RNAi, the situation becomes even more complex with persistent-propagative transmitted plant viruses, e.g., orthotopoviruses, tenuiviruses, and emaraviruses [110], due to the possibility of a bi-directional trans-kingdom RNAi between plant and insect vector (virus-induced and altered sRNA profiles in both species). Altered profiles from the insect vector will be transferred to healthy plants upon inoculation feeding by viruliferous insects and subsequently may alter the host plants’ physiology and metabolism differently compared to non-propagative transmitted viruses. During this process of inoculation access feeding by these insect vectors on healthy plants, protein effectors such as VSRs, produced in the insect vector during virus propagation, may come along as well and help to repress defense responses and rapidly establish viral infections in the recipient host. Whether some of these effects are unique and absent from plants inoculated with nonpersistently transmitted plant viruses remains to be answered.

Considering our current knowledge on antiviral RNAi, studies on trans-kingdom RNAi between plant host, virus, and insect vectors will be one of the next challenges for the future. It will not only provide a much more integral and holistic view and help us to further decipher their tritrophic interplay and communication, but also will contribute to our understanding on the (protein and RNA) languages spoken and in specific, the importance of specific sRNAs in these. Their identification may provide new targets for the development of strategies that may help to either prime/boost host plant defense responses and increase the resilience of crops against biotic stressors, or also interfere with virus transmission and the competence of insect vectors.

## Figures and Tables

**Table 1:** Studies that have demonstrated evidence of plant virus-derived trans-acting sRNAs with implicated roles in the viral–host interplay.

Virus	Studied Host	Target	Implicated Role	Reference
Cucumber mosaic virus (CMV) Y satellite RNA (Y-sat)	*Nicotiana tabacum*	Magnesium protoporphyrin chelatase subunit I gene (ChlI)	Disorder in chlorophyll synthesis; yellowing of the leaves	[32,33]
Tobacco mosaic virus (TMV)	*Nicotiana benthamiana*	C2-domain abscisic acid (ABA)-related (CAR) 7-like protein	Interfere with the levels of abscisic acid (ABA)	[35]
Chinese wheat mosaic virus (CWMV)	*Triticum aestivum*	Vacuolar (H^+^)-PPases (VPs)	Suppression of cell death induced by VPs	[36]
Rice stripe tenuivirus (RSV)	*Nicotiana benthamiana;* Rice	Several chloroplast-related genes; Eukaryotic translation initiation factor 4A (eIF4E)	Leaf-twisting and stunting; activation of antiviral autophagy	[37,38]
Wheat yellow mosaic virus (WYMV)	Wheat	Thioredoxin-like gene (TaAAED1)	Interfere with the scavenging of reactive oxygen species (ROS)	[39]

**Table 2:** Studies describing plant biological processes potentially and mostly affected by virus-derived sRNA-mediated gene deregulation.

Plant Biological Process	Virus-Derived sRNA	Potential Modulatory Effect	Reference
Stress responses	vsiRNAs; vmiRNAs	Decrease expression of stress resistance genes leading to increased susceptibility of the host to other (a)biotic stimuli.	[11,40,41,42]
Protein synthesis	vsiRNAs	Decrease expression of plant proteins involved in the antiviral response.	[40,43]
Chloroplast functions	vsiRNAs	Induce development of leaf-associated disease symptoms	[41,44]
Development	vsiRNAs	Modulate genes involved in pollen or anatomical structure development.	[41,45]
Cellular structure	vsiRNAs	Modulate expression of cellular structural proteins, such as kinesins and ribosomal proteins, interfering with general aspects of the cellular functioning.	[46,47,48]
Cell metabolism	vsiRNAs; vmiRNAs	Modulate expression of enzymes involved in biosynthesis of secondary metabolites, amino acids, starch and sucrose metabolism, and carbon metabolism.	[42,46,47,48,49,50,51]
Host defense pathways	vsiRNAs; vmiRNAs	Modulate expression of transcription factors (TFs), nucleotide binding site–leucine-rich repeats (NBS LRR), and receptor-like protein (RLP) kinase involved in host defense pathways.	[11,41,43,45,46,47,49,52]
Host RNA silencing pathway	vsiRNAs	Modulate expression of RNA silencing-associated factors (e.g., DCLs and AGO) involved in antiviral RNA-based response.	[41]
Synergistic infection	vsiRNAs; vmiRNAs	Modulate pathways to enhance synergistic infection and virulence of multiple viruses.	[41,50,53]

**Table 3:** Studies reporting viral siRNAs (vsiRNAs), (differentially expressed) viral-activated miRNAs (vamiRNAs), and viral-activated host encoded siRNAs (vasiRNAs) targeting host genes, and their implicated role in the viral–host interplay.

Virus (and Inducer)	vamiRNAs	Host	Targets	Implicated Role	Reference
CymRSV (p19), CrTMV, PVX, TEV, TCV, RMV, SHMV	miRNA168	*Arabidopsis thaliana*, *N. benthamiana*, *Medicago truncalata*, *Solanum lycopersicum*	AGO1	Translational repression of AGO1; alleviates antiviral RNAi pressure, and benefits viral infections.	[61]
PVY	miRNAs	*N. tabacum*	Genes from a broad range of cellular processes	Regulation of plant resistance to PVY.	[64]
PVY	miRNAs	*S. lycopersicum*	R genes, MAPKs and disease-responsive genes	A functional role of sRNA-mediated defenses in the recovery phenotype of tomato.	[67]
RSV	miRNA444	*Oryza sativa*	MIKCC-type MADS box proteins OsMADS23, OsMADS27a, and OsMADS57	Relieve the repression of RDR1 by MADS box proteins.	[52]
TSWV	miRNAs	Capsicum	ND	ND	[68]
SrMV	miRNAs	Saccharum spp.	Various	Associated with early immune response upon virus infection.	[69]
**Virus (and Inducer)**	**vasiRNAs**	**Host**	**Targets**	**Implicated Role**	**Reference**
ORMV	miRNAs, ml-siRNA, vasiRNAs	*A. thaliana*	Various	At the time point of sRNA analysis, no major changes in RNA targets were observed.	[29]
CMV, TuMv	vasiRNAs	*A. thaliana*	Various; genes responsive to biotic and abiotic stimuli are significantly enriched	Broad spectrum viral defense.	[30]
CaMV	vasiRNAs	*A. thaliana, Brassica rapa, B. napus*	13 loci (targets) out of 15, from which vasiRNAs derive, perfectly match those from Cao et al. [30].	Loci/CDS targets encode factors involved in photosynthesis and stress response (e.g., Rubisco activase (RCA), senescence-associated protein, heat shock protein HSP70, light harvesting complex, and membrane-related protein CP5). During infection, the expression of these factors is significantly downregulated, suggesting that their silencing is a central component of the plant’s response to virus infections.	[62]
TSWV, CaCV	vasiRNAs	*Capsicum annuum* cv. Yolo Wonder, *N. benthamiana* LAB (RDR1-deficient), and *N. benthamiana* WA, Tomato Marglobe (Sw5^−^), Red Defender (Sw5^+^)	Similar to those reported by Cao et al. [30] and enriched for ribosomal protein-encoding genes and genes involved in protein processing at the endoplasmic reticulum.		[63]
TuMV	vasiRNAs	*B. napus* (local)	Host genes and viral sequences	vasiRNAs, hsiRNAs and vsiRNAs are observed to target host as well as vRNA molecules to potentially control gene homeostasis during virus infection, and therefore contributing to virus–host compatibility.	[26]

CymRSV, cymbidium ringspot virus; CrTMV, crucifer-infecting tobacco mosaic virus; PVX, potato virus X; TCV, turnip crinkle virus; RMV, ribbgrass mosaic virus; SHMV, Sunn-hemp mosaic virus; CMV, cucumber mosaic virus; TuMV, turnip mosaic virus; PVY, potato virus Y; CaMV, cauliflower mosaic virus; RSV, rice stripe virus; SrMV, sorghum mosaic virus; TSWV, tomato spotted wilt virus; CaCV, capsicum chlorosis virus; ND, not determined.

## Data Availability

Not applicable.
